# Hyperglycaemia disrupts conducted vasodilation in the resistance vasculature of db/db mice

**DOI:** 10.1016/j.vph.2018.01.002

**Published:** 2018-04

**Authors:** Hamish A.L. Lemmey, Xi Ye, Hong C. Ding, Christopher R. Triggle, Christopher J. Garland, Kim A. Dora

**Affiliations:** a Department of Pharmacology, University of Oxford, Mansfield Road, Oxford OX1 3QT, UK; b Department of Pharmacology, Weill Cornell Medicine in Qatar, P.O. Box 24144, Education City, Doha, Qatar

**Keywords:** Cx, connexin, EC, endothelial cell, EDH, endothelium-derived hyperpolarization, GJ, gap junction, SK_Ca_, small-conductance Ca^2 +^-activated potassium channel, IK_Ca_, intermediate-conductance Ca^2 +^-activated potassium channel, PAR2, proteinase-activated receptor 2, SLIGRL, Ser-Leu-Ile-Gly-Arg-Leu peptide, VSMC, vascular smooth muscle cell, Resistance arteries, Hyperglycaemia, Db/db mice, EDH, Conducted vasodilation

## Abstract

Vascular dysfunction in small resistance arteries is observed during chronic elevations in blood glucose. Hyperglycaemia-associated effects on endothelium-dependent vasodilation have been well characterized, but effects on conducted vasodilation in the resistance vasculature are not known. Small mesenteric arteries were isolated from healthy and diabetic db/db mice, which were used as a model of chronic hyperglycaemia. Endothelium-dependent vasodilation via the G_q/11_-coupled proteinase activated receptor 2 (PAR2) was stimulated with the selective agonist SLIGRL. The Ca^2 +^-sensitive fluorescent indicator fluo-8 reported changes in endothelial cell (EC) [Ca^2 +^]_i_, and triple cannulated bifurcating mesenteric arteries were used to study conducted vasodilation. Chronic hyperglycaemia did not affect either EC Ca^2 +^ or local vasodilation to SLIGRL. However, both acute and chronic exposure to high glucose or the mannitol osmotic control attenuated conducted vasodilation to 10 μM SLIGRL. This investigation demonstrates for the first time that a hypertonic solution containing glucose or mannitol can interfere with the spread of a hyperpolarizing current along the endothelium in a physiological setting. Our findings reiterate the importance of studying the effects of hyperglycaemia in the vasculature, and provide the basis for further studies regarding the modulation of junctional proteins involved in cell to cell communication by diseases such as diabetes.

## Graphical abstract

**Figure:**
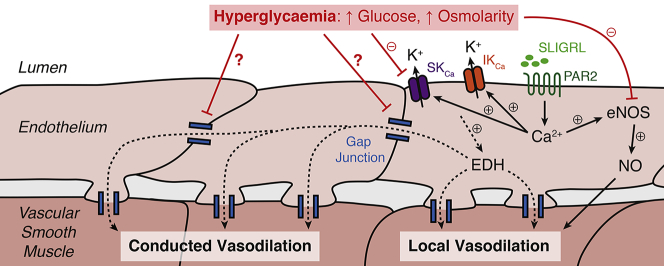

## 1 Introduction

Small resistance arteries isolated from animal models of diabetes and obesity exhibit an altered sensitivity to activators of endothelium-dependent vasodilation, including acetylcholine (ACh), bradykinin, and the proteinase-activated receptor 2 (PAR2) ligand Ser-Leu-Ile-Gly-Arg-Leu peptide (SLIGRL) [1], [2], [3], [4], [5], [6]. Endothelial cell (EC) dysfunction is a hallmark of chronic hyperglycaemia and has been attributed to a loss of nitric oxide (NO) synthesis and bioavailability [6], [7], [8], structural remodeling [9], increased generation of reactive oxygen species (ROSs) [10], [11], and, most relevant to the current investigation, altered endothelium-dependent hyperpolarization (EDH) [2], [12], [13], [14], [15].

EDH contributes significantly to endothelium-dependent vasodilation in the resistance vasculature. Following mobilisation of EC Ca^2 +^, EDH is generated by an efflux of K^+^ through small- and intermediate-conductance Ca^2 +^-activated potassium channels (SK_Ca_, IK_Ca_), which are found in ECs and not the adjacent smooth muscle. The hyperpolarizing signal then spreads to neighbouring vascular smooth muscle cells (VSMCs) closing L-type Ca^2 +^ channels leading to smooth muscle relaxation and an increase in artery diameter [16], [17], [18]. Furthermore, EDH can spread rapidly between neighbouring ECs via gap junctions (GJs) to stimulate VSMC relaxation distant to the origin of the hyperpolarizing signal [19], [20]. This phenomenon, termed conducted vasodilation, maximizes the response of an artery to a vasodilatory signal and plays a significant role in the regulation of vascular tone and blood flow [18]. This helps to ensure that resistance and flow upstream drop sufficiently to increase blood flow.

There is a large amount of literature on the deleterious effects of hyperglycaemia on endothelium-dependent vasodilation. Lagaud et al. [7] reported diminished ACh-mediated vasodilation of mesenteric arteries isolated from db/db mice, but, interestingly, increased vasodilation when cyclooxygenase and NO synthesis were inhibited. The increase in vasodilation was explained by loss of constrictor prostanoid and enhanced EDH in the absence of NO, respectively. Other studies with resistance arteries from the db/db mouse model reported reduced vasodilation to ACh accompanied by abnormal EC Ca^2 +^ signaling [5] or a greater dependence on L-NAME-insensitive pathways [8]. Previous studies conducted on other models of hyperglycaemia have, in addition, identified components of EDH which may be altered during hyperglycaemia. Burnham et al. [14] used the Zucker diabetic fatty rat model and measured changes in mesenteric VSMC membrane potential. They reported a reduction in EDH, specifically in the SK_Ca_-mediated component. Experiments with middle cerebral arteries from rats fed a high-fat diet have suggested large-conductance calcium-activated potassium channels on VSMCs are also perturbed during hyperglycaemia [4].

Collectively, these findings provide strong evidence for hyperglycaemia exerting a significant effect on EC-mediated vasodilation. This will then contribute to the detrimental effect of diabetes on the cardiovascular system. In spite of the extensive evidence probing the mechanism by which hyperglycaemia disrupts endothelium-dependent vasodilation, the impact it may have on conducted vasodilation has not been reported. Conducted vasodilation is a crucial physiological response ensuring a coordinated change in blood flow within the microcirculation. In mouse mesenteric arteries, robust conducted vasodilation can be evoked using SLIGRL, and is not dependent on the production of NO, but is associated with local and conducted hyperpolarization [21], [22], [23], [24]. Since in our hands EDH responses to ACh are weak [24], [25], [26], the current investigation tested the hypothesis that endothelial dysfunction in resistance arteries following acute and chronic hyperglycaemia will result in attenuated conducted vasodilation mediated by SLIGRL.

## 2 Materials and methods

### 2.1 Animal model

All animal procedures were conducted in accordance with the University of Oxford local ethical guidelines, the ARRIVE guidelines, and the Animal (Scientific Procedures) Act, 1986. 11–18-week-old db/db (BKS·Cg-Dock7^m^ +/+Lepr^db^J) mice, which lack a functional leptin receptor, were used as a model of diabetes and chronic hyperglycaemia. Healthy age-matched wild-type (C57BL/6NCrl) mice (Charles River, UK) were used in control experiments. Animals were sacrificed by rising CO_2_ and confirmed with cervical dislocation (as specified by Schedule 1 of the Animals [Scientific Procedures] Act 1986, UK).

### 2.2 Blood glucose measurement and tissue collection

Blood glucose level was measured with an AlphaTRAK 2 Blood Glucose Monitoring System (Zoetis, Parsippany, NJ, USA). A blood sample was obtained from the heart in 0.4% sodium citrate and centrifuged at ~ 24 g for 5 min. The isolated plasma was collected and its osmolarity measured with a Roebling Micro-Digital Osmometer (Camlab, Cambridge, UK). The original plasma osmolarity was calculated after correction for the sodium citrate in the sample. The mesentery was excised and placed in ice-cold 3-(N-morpholino)propanesulfonic acid (MOPS)-buffered solution containing (in mM): 142.5 NaCl, 4.7 KCl, 1 CaCl_2_(2H_2_O), 1.17 MgSO_4_(7H_2_O), 3 MOPS, 1.2 (H_2_O)NaH_2_PO, 2 pyruvate, 0.02 EDTA, 3.44 NaOH, 11 (for WT) or 40 (for db/db) glucose, with pH adjusted to 7.40 ± 0.02 with NaOH. The high glucose MOPS-buffered solution was used throughout all experimentation with arteries isolated from db/db mice. 11 and 40 mM glucose represent the non-fasting blood glucose levels previously reported in WT and db/db mice, respectively [27], [28].

### 2.3 Artery isolation and local vasodilation measurement

Second or third-order segments of mesenteric arteries were isolated from connective tissue, cannulated with two glass micropipettes in a temperature-regulated chamber (2 mL, Warner Instruments, CT, USA), and placed on the stage of an inverted microscope (FluoView1000 linescan confocal, Olympus, Tokyo, Japan) as previously described [24], [29]. The preparation was continuously superfused with MOPS-buffered solution (2 mL/min), warmed to 36–37 °C, and pressurized to 60 mmHg with a custom-built gravity-fed inflow and outflow system. Arteries were visualized using a 10 ×/0.3 Olympus objective and images recorded with FluoView software (Olympus, Tokyo, Japan). Vascular reactivity was assessed by sub-maximal vasoconstriction with 1–3 μM phenylephrine (PE) and endothelium-dependent relaxation to 10 μM SLIGRL. Only arteries which dilated to 90% of the passive diameter were used further. 100 μM N^G^-nitro-L-arginine methyl ester (L-NAME), 1 μM TRAM-34, and 100 nM apamin were added to the superfusion, whilst all other agents were added to the bath directly. The cumulative addition of L-NAME, TRAM-34, apamin, and 45 mM K^+^ was tested in all arteries.

### 2.4 EC Ca^2 +^ imaging

ECs of cannulated arteries were selectively loaded with a cell-permeable fluorescent Ca^2 +^ indicator to visualize Ca^2 +^ events. Briefly, intraluminal pressure was lowered to ~ 10 mmHg and the lumen was perfused for ~ 20 min with MOPS-buffered solution containing 0.0625% Pluronic F-127 and 12 μM fluo-8, AM. After the dye was washed out, the artery was re-pressurized to 20 mmHg and left for 15 min to allow de-esterification of the reporter dye. The artery was excited at ~ 488 nm and emitted light collected at ~ 505 nm with a 40 ×/1.15 water-immersion Olympus objective. Recordings of EC Ca^2 +^ events were acquired at a scan frequency of ~ 3 Hz.

### 2.5 Measurement of conducted vasodilation

Mesenteric arteries with a bifurcation (consisting of a feed artery and two side-branches) were isolated and cannulated with three glass micropipettes on the stage of an inverted microscope (FV1200, linescan confocal, Olympus, Tokyo, Japan) as described previously [24], [30]. In this way, in addition to an inflow and outflow cannula, a side-branch was cannulated to act as a delivery route for SLIGRL. Following the function test, the artery was preconstricted with PE and 10 μM SLIGRL was perfused into the side-branch for at least 2 min at 50 μL/min using a syringe pump system (Bioanalytical systems, IN, USA). Perfusion of the side-branch with SLIGRL resulted in local and conducted vasodilation upstream along the length of the feed artery. 0.1 μM 6-carboxyfluorescein was included with the SLIGRL solution to monitor movement of the side-branch perfusion solution. The artery was excited at ~ 488 nm and brightfield and fluorescence images simultaneously collected at ~ 505 nm with a 4 ×/0.16 Olympus objective. Some arteries were intraluminally perfused with solution containing 40 mM glucose or 29 mM mannitol + 11 mM glucose for 15 min immediately before delivering SLIGRL into the side-branch. The addition of mannitol in the solution increased the osmolarity to match the observed plasma osmolarity of db/db mice.

### 2.6 Drugs and solutions

All drugs were obtained from Sigma-Aldrich (Dorset, UK) with the exception of SLIGRL (Auspep, Victoria, Australia), apamin (Latoxan, Valence, France), fluo-8, AM (AAT Bioquest, CA, USA), and Pluronic F-127 (Molecular Probes, OR, USA). All stock solutions were prepared in distilled water except for TRAM-34, fluo-8, AM, and Pluronic F-127, which were dissolved in dimethyl sulfoxide. Prior to use, all drugs were diluted in MOPS-buffered solution. Inhibitors were incubated with the tissue for at least 30 min, except for apamin which was incubated for 1 h.

### 2.7 Statistical analysis

All data analysis was completed post-hoc with MetaMorph software (v7.7.4.0, Molecular Devices, CA, USA). In the case of conducted dilation, multiple, calibrated sites along the feed artery wall could be viewed and analyzed at the same time point, allowing direct comparison of local vasodilation (of the side-branch) to conducted vasodilation for a single application of SLIGRL. Conducted vasodilation experiments were analyzed at the time point where the peak, maximum response at the 0 mm position in the feed artery was obtained. This 0 mm site represented the first site upstream of the bifurcation where SLIGRL did not directly stimulate the cells. The data were then normalized to the time point where 50% of maximum diameter was obtained at the 0 mm position. The normalization accounts for any differences in response at the 0 mm site, thereby enabling comparisons regarding the rate of decay towards the 2.5 mm position upstream. 50% was chosen as it was the highest percentage were all data could be included. The vasodilation evoked by SLIGRL was calculated as the percentage of maximum diameter from PE-preconstriction. PE-preconstriction was calculated as a percentage of minimum diameter (0 μm) from the passive diameter. Average fluorescence intensity of an entire cell (F) was measured for a 30 s period approximately 20 s after SLIGRL application, in 6–13 cells per artery to provide one n value. Fluorescence was then normalized to the baseline fluorescence intensity (F_0_) to give F/F_0_. Dose-response curves, histograms, and graphs were prepared with Prism software (v7.0, GraphPad Software, CA, USA). Results are presented as mean ± SEM and sample size is indicative of the number of animals. Statistical comparisons were made using one-way or two-way ANOVA, where appropriate, with Bonferroni post-test for multiple comparisons. p < 0.05 was considered statistically significant.

## 3 Results

### 3.1 Animal model of diabetes phenotype

**Fig. 1:**
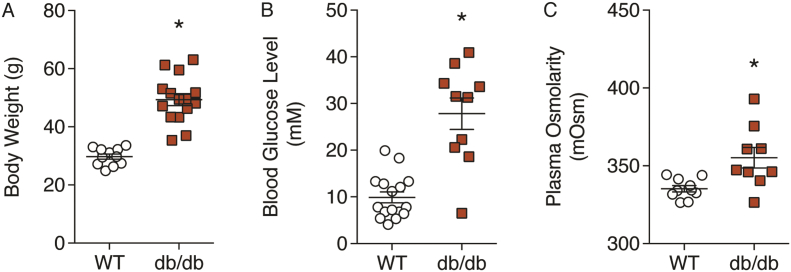
Animal characteristics. The phenotype of db/db mice was assessed by measuring body weight (A), blood glucose level (B), and plasma osmolarity (C). n = 9–16. *Denotes statistical significance compared to WT.

Diabetic db/db mice had a greater body weight, blood glucose level, and plasma osmolarity compared with WT mice (Fig. 1), confirming the leptin receptor mutation is associated with chronic hyperglycaemia.

### 3.2 SLIGRL-induced local vasodilation

Local vasodilation of isolated mesenteric arteries was assessed following submaximal vasoconstriction with 1–3 μM PE. This preconstriction was consistent between strains (WT: 43.5 ± 0.9%; db/db: 42.6 ± 1.9%). Passive (maximal) diameter also did not significantly differ between strains (WT: 224.4 ± 12.1 μm; db/db: 238.8 ± 6.1 μm).

**Fig. 2:**
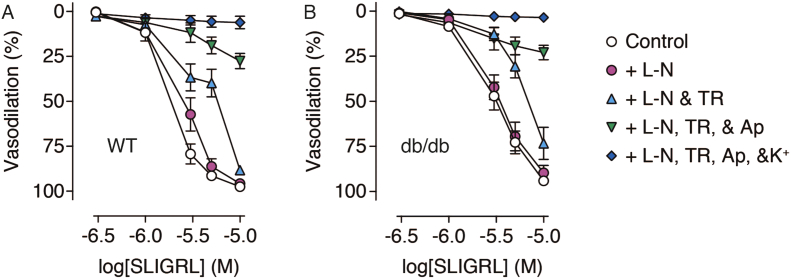
Concentration-response curves of SLIGRL-induced vasodilation in pressurized mesenteric arteries from WT (A) and db/db (B) mice. SLIGRL stimulated endothelium-dependent vasodilation which was insensitive to blockade of NO synthase with L-NAME (L-N). IK_Ca_ and SK_Ca_ were selectively blocked with TRAM-34 (TR) and apamin (Ap), respectively, which attenuated the response. Increasing extracellular K^+^ concentration to 45 mM (K^+^) completely abolished any dilatory response. n = 7–9.

**Fig. 3:**
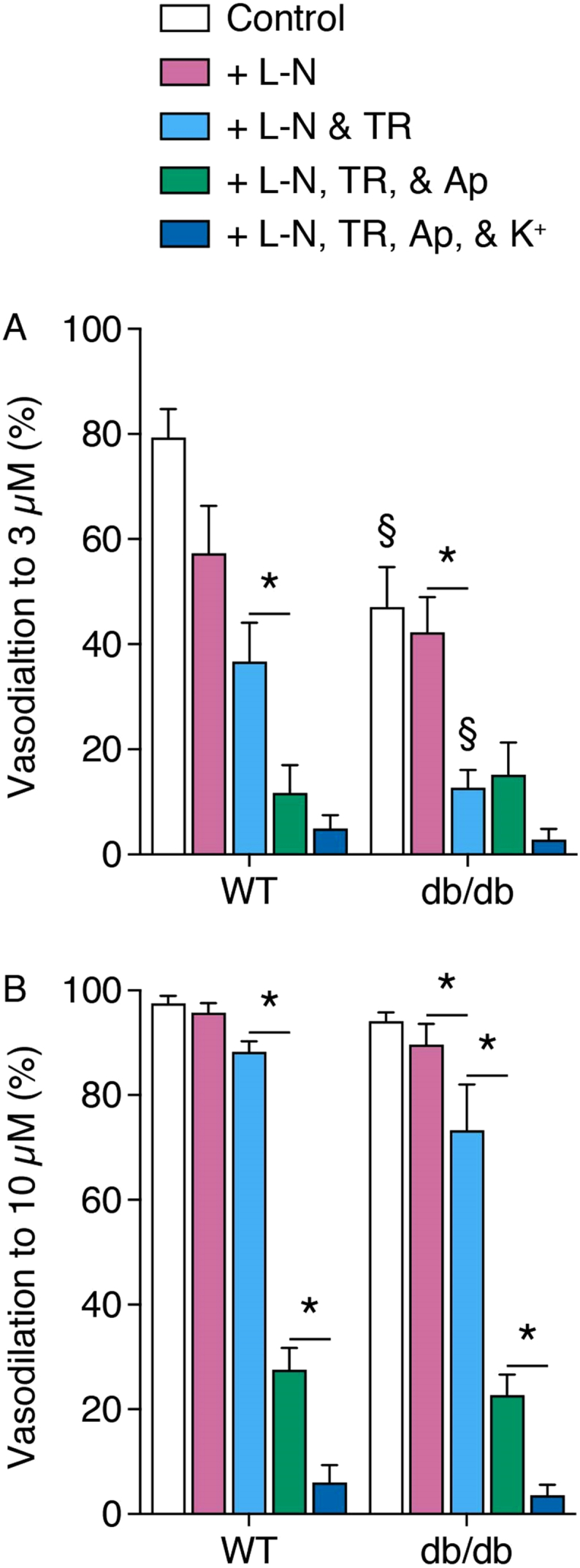
Bar charts of vasodilation stimulated by a submaximal (A) and maximal (B) concentration of SLIGRL in pressurized mesenteric arteries isolated from WT and db/db mice. L-N, L-NAME; TR, TRAM-34; Ap, apamin; K^+^, 45 mM K^+^. n = 7–9. *Denotes statistical significance between treatments. § denotes statistical significance compared to the same treatment in WT.

3 μM SLIGRL stimulated submaximal vasodilation, which was significantly greater in WT arteries than db/db arteries. The response to 10 μM SLIGRL, which stimulated maximum vasodilation, was the same between strains (Fig. 2, Fig. 3). To isolate the EDH component, 100 μM L-NAME was used to inhibit NO synthase, and did not attenuate vasodilation to either 3 or 10 μM SLIGRL. However, in the absence of NO synthesis, vasodilation to 3 μM SLIGRL was the same in both WT and db/db arteries (Fig. 2, Fig. 3).

Addition of the selective IK_Ca_ blocker TRAM-34 significantly reduced vasodilation to 3 μM SLIGRL in db/db arteries, but not WT arteries. TRAM-34 had no effect on vasodilation induced by 10 μM SLIGRL. In the additional presence of apamin, the selective SK_Ca_ blocker, vasodilation to 3 μM SLIGRL was reduced in WT arteries, and vasodilation to 10 μM SLIGRL was attenuated in both strains (Fig. 2, Fig. 3). In the continued presence of L-NAME, TRAM-34, and apamin, increasing extracellular K^+^ concentration to 45 mM produced no further blockade of vasodilation mediated by 3 μM SLIGRL. However, elevated extracellular K^+^ further attenuated vasodilation to 10 μM SLIGRL (Fig. 2, Fig. 3).

### 3.3 SLIGRL-induced EC Ca^2 +^ mobilisation

**Fig. 4:**
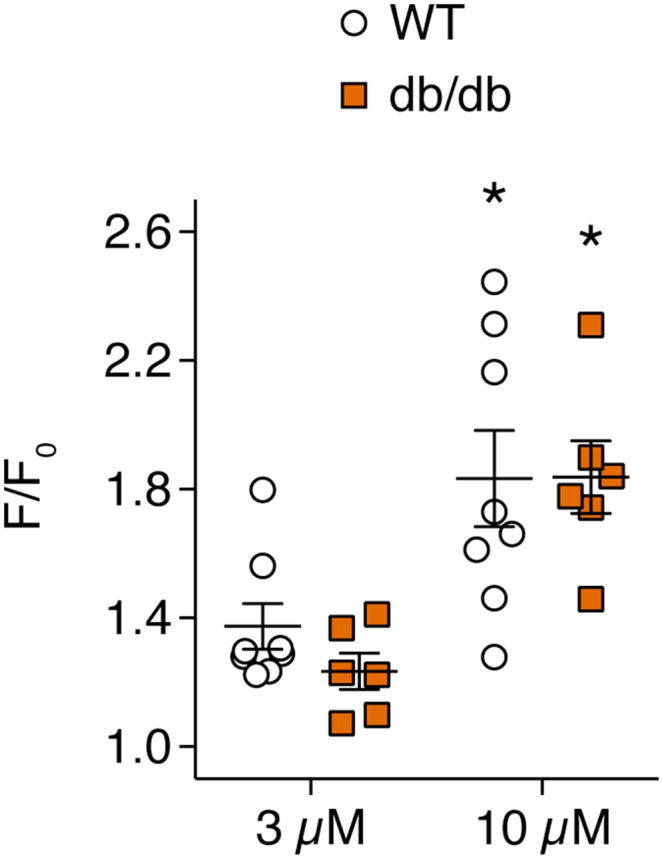
SLIGRL-induced Ca^2 +^ mobilisation in ECs of pressurized mesenteric arteries isolated from WT and db/db mice. ECs were selectively loaded with the Ca^2 +^-sensitive fluorescent indicator fluo-8. n = 6–8. *Denotes statistical significance compared to 3 μM SLIGRL within the same strain.

NO- and EDH-mediated vasodilation to SLIGRL is dependent on increases in EC Ca^2 +^. To visualize changes in EC Ca^2 +^ activity, ECs were selectively loaded with the Ca^2 +^-sensitive fluorescent indicator fluo-8. In response to 3 μM SLIGRL, EC Ca^2 +^ activity increased in arteries from WT and db/db mice (Fig. 4). 10 μM SLIGRL stimulated a further increase in EC Ca^2 +^ mobilisation in WT and db/db arteries. No difference was detected between strains at 3 or 10 μM SLIGRL (Fig. 4).

### 3.4 SLIGRL-induced conducted vasodilation

Following activation of PAR2 and increases in EC Ca^2 +^, the K_Ca_ channel-associated endothelium-dependent hyperpolarizing signal spread longitudinally along the endothelium to stimulate conducted vasodilation, as previously observed [24]. In some arteries, high glucose or the hyperosmolar control, mannitol, were perfused into the feed artery prior to the perfusion of SLIGRL to establish the effect of acute exposure of arteries to hyperglycaemia. The local vasodilation to 10 μM SLIGRL in the side-branch was not different between strains, or following acute exposure to a high glucose or hyperosmotic solution, and was only reduced in the combined presence of L-NAME and elevated extracellular K^+^ (WT Control: 95.5 ± 0.5%, n = 11; WT 40 mM glucose: 94.6 ± 0.8%, n = 5; WT + 29 mM mannitol: 94.2 ± 1.9% n = 6; db/db 40 mM glucose: 88.2 ± 2.8%, n = 5; db/db 40 mM glucose + L-NAME: 92.8 ± 1.5%, n = 5; db/db 40 mM glucose L-NAME + 45 mM K^+^: 22.5 ± 1.8%, n = 5).

**Fig. 5:**
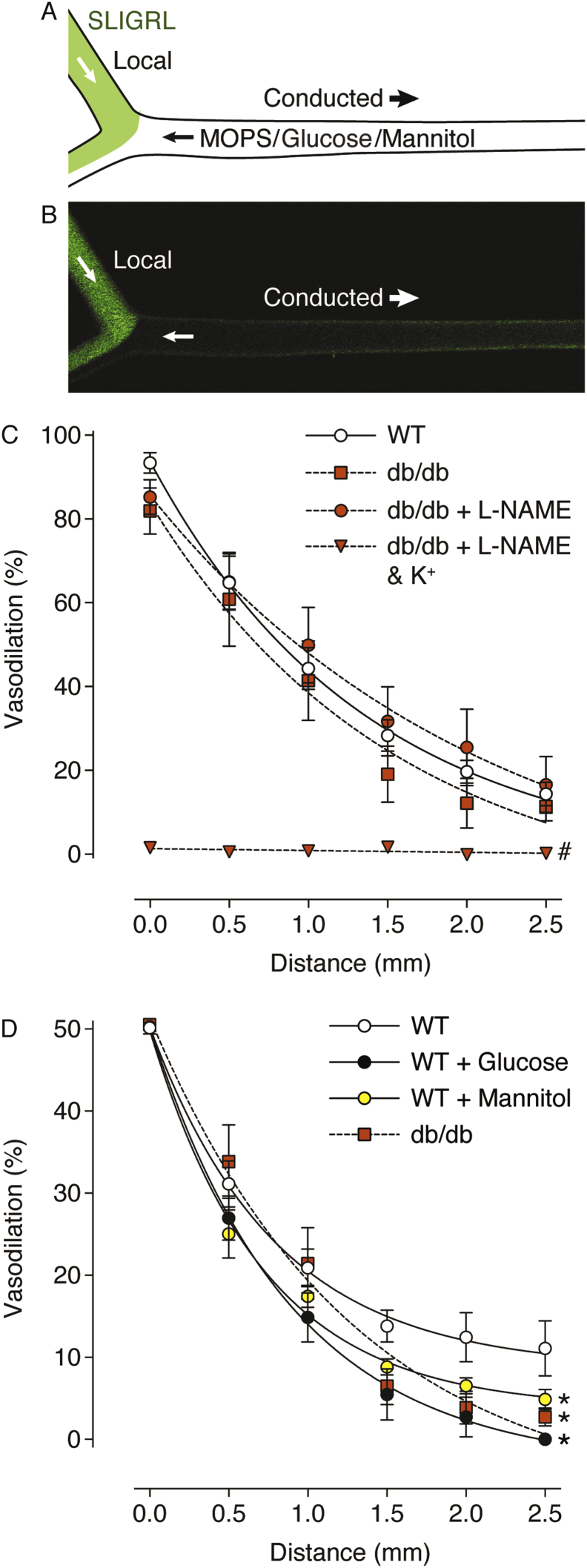
SLIGRL-induced conducted vasodilation along the feed artery of mesenteric artery bifurcations isolated from WT and db/db mice. Schematic (A) and confocal fluorescence micrograph (B) of experimental setup and delivery of 10 μM SLIGRL (green) to the downstream end of the artery via the bifurcation. The artery is aligned with sites (distance, mm) for diameter values used to calculate percentage vasodilation in (C) and (D). Arteries from db/db mice were incubated with L-NAME and 45 mM K^+^. Some arteries from WT mice were also perfused with 40 mM glucose or 29 mM mannitol prior to SLIGRL perfusion. Data are expressed as the peak (C) and normalized (D) responses. By normalizing data to 50% dilation at 0 mm, more subtle differences related to the decay of dilation with distance from the bifurcation were observed, n = 5–11. # denotes statistical significance compared to WT, db/db, and db/db + L-NAME. * denotes statistical significance compared to WT control. (For interpretation of the references to colour in this figure legend, the reader is referred to the web version of this article.)

Under control conditions, conducted vasodilation was observed along the feed artery, exponentially decaying with distance, and was the same in arteries from WT and db/db mice (Fig. 5). As previously shown in arteries from WT mice [24], the spread of vasodilation in the arteries from db/db mice was insensitive to L-NAME, but completely abolished by increasing extracellular K^+^ (Fig. 5). The dilation at the 0 mm position was more variable in the presence of mannitol and glucose, precluding accurate comparisons between data sets. Therefore, to compare the decay of dilation with distance upstream along the feed artery, responses upstream (0 through 2.5 mm) were normalized to 50% dilation at 0 mm. In this more sensitive method of comparing data it was observed that conducted vasodilation decayed more rapidly following luminal exposure to hyperosmolar solutions. The vasodilation of WT arteries when measured at the 2.5 mm site was reduced > 10-fold during acute exposure to a high glucose-containing solution (Fig. 5). Conducted vasodilation was also attenuated in arteries exposed to hypertonic mannitol, and in arteries from db/db mice. At the 2.5 mm site, vasodilation was ~ 3 fold greater in WT arteries than db/db arteries (Fig. 5). This difference was not apparent when looking at the maximum response (Fig. 5C), only when normalized (Fig. 5D).

## 4 Discussion

The aim of the current investigation was to characterize the effect hyperglycaemia may have on endothelium-dependent responses, namely EDH-mediated local and conducted vasodilation in the resistance vasculature. Previous studies with mesenteric resistance arteries from the diabetic db/db mouse model have reported conflicting results as far as EDH-mediated vasodilation is concerned. Furthermore, there has been no previous report of the possible impact of diabetes on conducted vasodilation. Our results demonstrate for the first time that SLIGRL-induced conducted vasodilation in the resistance vasculature of db/db mice is compromised, which may help explain the detrimental effects of diabetes on the microcirculation and the tissue.

In our preparation, the inhibition of NO synthesis did not significantly affect vasodilation, suggesting EDH is more important for SLIGRL-mediated responses or can compensate for NO loss. This is also consistent with the finding that hyperpolarization is crucial for conducted vasodilation [24], [31]. Local vasodilation to 3 μM SLIGRL was significantly lower in db/db arteries than WT arteries. During NO inhibition, however, no difference between strains was observed, suggesting only the L-NAM*E*-sensitive component is attenuated. On the other hand, vasodilation to a maximal concentration of SLIGRL under control conditions was the same between healthy and diabetic arteries. One possibility is the degree of EC Ca^2 +^ mobilized by 10 μM SLIGRL is enough to activate a sufficient EDH response to overcome the loss of NO-dependent vasodilation. Loss of NO synthesis has been well-characterized in the db/db mouse model [7], [8], [11] and other rodent models of diabetes and hyperglycaemia [2], [4], [32]. There is, however, considerable controversy in the literature regarding EDH production in the db/db mouse.

Here, we failed to observe any significant difference between WT and db/db arteries in the presence of L-NAME, supporting the notion that EDH-dependent vasodilation is maintained [4], [7]. We did, however, see a difference in vasodilation to 3 μM SLIGRL between strains in the additional presence of TRAM-34. This may reflect a loss of SK_Ca_ function during chronic hyperglycaemia, which was also observed by Burnham et al. [14] in the Zucker diabetic fatty rat and Ding et al. [1] in the streptozotocin-induced apolipoprotein E-deficient diabetic mouse. The latter reported a reduction in SK_Ca_ gene expression, as did Ma et al. [3] in the streptozotocin-induced diabetic rat. These studies concluded SK_Ca_ is essential for mediating EDH during endothelium-dependent vasodilation and its function may be perturbed during hyperglycaemia; both claims are supported by our findings. In db/db arteries, vasodilation to 10 μM SLIGRL was also sensitive to TRAM-34 but not significantly different to the response induced in WT arteries. Again, this may be because the EC Ca^2 +^ response stimulated by a maximal concentration of SLIGRL can stimulate enough IK_Ca_ activity to mask the loss of SK_Ca_.

In the current study, we observed no significant difference between SLIGRL-induced changes in Ca^2 +^ activity in ECs from WT and db/db arteries (Fig. 4). This result supports the notion that attenuated vascular reactivity reported in hyperglycaemia is due to Ca^2 +^-independent processes such as uncoupling of endothelial NO synthase leading to ROS generation and, as a result, diminished NO production [33] and enhanced contractility [34], [35]. The lack of effect of hyperglycaemia on EC Ca^2 +^ can also explain how EDH-dependent vasodilation is maintained. However, this outcome is in direct disagreement with findings from Chen et al. [5]. They reported a reduction in ACh-mediated EC Ca^2 +^ activity in db/db mesenteric arteries, specifically the number of ECs which responded and the relative change in calcium-sensitive indicator fluorescence. Our use of SLIGRL rather than ACh as a mobilizer of EC Ca^2 +^ may explain this discrepancy, as they also observed more pronounced differences in local vasodilation between healthy and diabetic arteries, and in our hands the vasodilation to ACh is more dependent on NO [24], [25], [26].

Luminal perfusion of 10 μM SLIGRL stimulated conducted vasodilation 2.5 mm away from the site of local vasodilation. By using 10 μM SLIGRL, the local dilation was maximal, and not different between strains or treatments. This also allowed submaximal values to be compared, during the onset of dilation, normalized to 50% dilation at the 0 mm site in the feed artery. The response at this 0 mm site is already upstream from direct stimulation with SLIGRL, is not affected by inhibitors of NOS, and is associated with hyperpolarization [24]. Therefore by comparing these submaximal responses, we were able to uncover subtle differences in the conducted dilation responses. Conducted vasodilation of arteries from db/db mice persisted in the presence of NO blockade by L-NAME, but was completely abolished following exposure to elevated extracellular K^+^, confirming the phenomenon is dependent on the generation of EDH, as previously described in healthy young mice (Fig. 5) [24].

Acute perfusion of a high glucose solution for 15 min beforehand attenuated conducted vasodilation, as did perfusion of a mannitol-containing solution with the same tonicity as the high glucose solution. This result suggests the increased osmolarity of the high glucose solution could be responsible for the reduced conduction, rather than a glucose-specific effect. Previously, experiments performed on cultured VSMCs from rat tail arteries exhibited similar alterations in Ca^2 +^ activity following exposure to a solution containing elevated glucose or mannitol [36]. However, glucose-specific effects of hyperglycaemia have previously been reported in endothelium-dependent increases of human forearm blood flow [37], ACh-mediated vasodilation of isolated rabbit basilar arteries [38], and GJ permeability in cultured bovine aortic VSMCs [39]. Thus, there is evidence for and against glucose-specific effects in the vasculature during hyperglycaemia, and our findings lend more support to the latter.

EDH-dependent conducted vasodilation has been shown to be disrupted in mouse models of hypercholesterolaemia [40] and aging [41]. Our investigation adds diabetes to this list as conducted vasodilation in arteries from db/db mice was significantly less than in the WT arteries, at least when normalized to a submaximal response, a more sensitive method of detection. Interestingly, the maximal response to 10 μM SLIGRL was the same in WT arteries bathed in 11 mM glucose and the db/db arteries bathed in 40 mM glucose. This suggests a form of compensation by this chronic exposure to elevated glucose in the db/db mice. Not only does the effect of hypertonic solution appear to be compensated, but also the effect of glucose. Note also that we deliberately measured the levels of glucose and osmolarity in the db/db mice and mimicked these conditions during collection, dissection and performing experiments, which is not usually performed by others, but is perhaps more appropriate. Overall our data suggest that both acute and chronic hyperglycaemia elicit an effect on conducted responses, with acute exposure more detrimental than chronic exposure. Whether this is a physiological response has yet to be established, but could relate to post-prandial rest and digest responses.

The lack of effect of hyperglycaemia on local vasodilation and EC Ca^2 +^ activity suggests the reduced conducted vasodilation is due to attenuated upstream spread of the hyperpolarizing signal between ECs, which could reflect the greater dissipation of hyperpolarizing current through plasmalemmal ion channels, and/or the reduced electrical coupling between homo and/or heterocellular GJs. Regardless of the mechanism, the ability for a given hyperpolarizing current to pass upstream appears reduced with hyperglycaemia. Although this is the first study to show conducted vasodilation is reduced by hyperglycaemia, it is not the only investigation to report conducted vasomotor responses in a mouse model of diabetes. Rai et al. [42] observed attenuated conducted vasoconstriction in cremasteric arterioles of streptozotocin-induced diabetic mouse, which could be rescued with a combination of protein kinase C β inhibition and ROS scavenging. Activation of protein kinase C-dependent signaling by chronic high glucose has previously been shown to reduce transfer of dye by GJs in cultured microvascular ECs from rat retinal arteries. This effect was accompanied by a downregulation of Cx43 gene expression [43]. In mesenteric arteries from streptozotocin-induced diabetic apoE-deficient mice, on the other hand, a reduction in Cx37 expression rather than Cx43 expression was observed; whereas no difference was detected for mRNA expression levels of connexins 37, 40, 43 and 45 in small mesenteric arteries from db/db versus healthy controls [28]. However, both Cx37 and Cx43 proteins are postulated to play a pivotal role in EC-EC and VSMC-VSMC coupling, and the movement of EDH through myoendothelial GJs to stimulate vasodilation [44], [45], [46]. Furthermore, pre-treatment with a selective inhibitor of protein kinase C β prevented impairment of endothelium-dependent vasodilation by acute hyperglycaemia in arteries of the human forearm [47]. The authors concluded activation of protein kinase C β occurs rapidly during hyperglycaemia and contributes to the onset of endothelial dysfunction. Together with the findings of previous studies, our investigation suggests the effect of hyperglycaemia on protein kinase C-dependent regulation of Cx function is a promising avenue for future research.

## 5 Conclusions

To summarize, the current investigation provides the first evidence that hyperglycaemia disrupts the ability for hyperpolarizing current to pass longitudinally along the length of arteries and stimulate conducted dilation in the resistance vasculature of db/db mice. Interestingly, both acute and chronic exposure to high glucose attenuated conducted vasodilation without affecting EDH-dependent local vasodilation or EC Ca^2 +^ mobilisation. We conclude hyperglycaemia affects the spread of EDH, thus interfering with the coordinated response of the microcirculation to a dilatory stimulus, which may have significant implications for diabetic patients.

## Funding

This research was supported by a British Heart Foundation PhD Studentship awarded to Hamish A.L. Lemmey, a British Heart Foundation project grant PG/14/58/30998, a Qatar Foundation National Priorities Research Program grant 4-9103-244.

## Conflicts of interest

The authors declare no conflicts of interest.
